# Testing Measurement Invariance across Groups of Children with and without Attention-Deficit/ Hyperactivity Disorder: Applications for Word Recognition and Spelling Tasks

**DOI:** 10.3389/fpsyg.2017.01891

**Published:** 2017-10-25

**Authors:** Patrícia S. Lúcio, Giovanni Salum, Walter Swardfager, Jair de Jesus Mari, Pedro M. Pan, Rodrigo A. Bressan, Ary Gadelha, Luis A. Rohde, Hugo Cogo-Moreira

**Affiliations:** ^1^Department of Psychology and Psychoanalysis, State University of Londrina, Londrina, Brazil; ^2^Department of Psychiatry, Federal University of São Paulo, São Paulo, Brazil; ^3^Department of Psychiatry, Federal University of Rio Grande do Sul, Porto Alegre, Brazil; ^4^National Institute of Developmental Psychiatry for Children and Adolescents, São Paulo, Brazil; ^5^Department of Pharmacology and Toxicology, University of Toronto, Toronto, ON, Canada

**Keywords:** measurement invariance, differential item functioning, word recognition, spelling, ADHD, group comparison

## Abstract

Although studies have consistently demonstrated that children with attention-deficit/hyperactivity disorder (ADHD) perform significantly lower than controls on word recognition and spelling tests, such studies rely on the assumption that those groups are comparable in these measures. This study investigates comparability of word recognition and spelling tests based on diagnostic status for ADHD through measurement invariance methods. The participants (*n* = 1,935; 47% female; 11% ADHD) were children aged 6–15 with normal IQ (≥70). Measurement invariance was investigated through Confirmatory Factor Analysis and Multiple Indicators Multiple Causes models. Measurement invariance was attested in both methods, demonstrating the direct comparability of the groups. Children with ADHD were 0.51 SD lower in word recognition and 0.33 SD lower in spelling tests than controls. Results suggest that differences in performance on word recognition and spelling tests are related to true mean differences based on ADHD diagnostic status. Implications for clinical practice and research are discussed.

## Introduction

Attention-deficit/hyperactivity disorder (ADHD) is a neurodevelopmental condition encompassing symptoms of inattention, hyperactivity, and impulsivity that interfere with a person’s daily functioning (DSM-5; American Psychiatric Association, 2013). The worldwide prevalence of ADHD is around 5% and there is evidence for stability of its prevalence estimates over the past three decades, even when considering different study methodologies (Polanczyk et al., 2007, 2014).

There is a clear association between ADHD and learning disabilities, with comorbidity rates ranging from 31 to 45% (e.g., DuPaul et al., 2013). Moreover, children and adolescents with ADHD often achieve lower test scores than their peers in academic areas including word recognition, reading comprehension, mathematical reasoning, and spelling/handwriting (e.g., Miller et al., 2013; Johnels et al., 2014; Martinussen and Mackenzie, 2015; Re and Cornoldi, 2015; Tosto et al., 2015; Pham, 2016). In their great majority, these studies have been based on direct comparisons of groups of ADHD children with controls in standardized measures on cognitive tasks.

However, comparing groups of children with/without ADHD in some psychological attribute or ability relies on the assumption that the tasks used to evaluate such constructs are, in fact, assessing equivalent constructs in each group. This assumption might not be true for several reasons. For example, attentional problems of children with ADHD could interfere in the development of the representation of the words in the lexicon, what in turn could produce differences in the construct representation of reading or spelling abilities. Therefore, it is necessary to empirically demonstrate if the observed scores in a measure represent the same latent trait for different subpopulations in which the test is used (e.g., subpopulations based on gender, race, etc.), and between which the test maybe compared statistically. Demonstrating this property, measurement invariance, is a prerequisite for valid comparisons between groups (Meredith, 1993), for example in any simple *t*-test or analyses of variance procedures (Vandenberg and Lance, 2000).

Word recognition and spelling under dictation are cognitive abilities related to the acquisition of mental representations of words, which depends heavily on phonological and orthographic skills (Nunes and Bryant, 2013). While reading single words aloud requires retrieval of the pronunciation of a given word from the mental lexicon (or gathering the pronunciation through sublexical components, such as morphemes), spelling single words under dictation involves the transformation of a spoken input into an orthographic form (Castles and Coltheart, 1993; Tainturier and Rapp, 2001). It should be considered that symptoms of ADHD might interfere with the development of phonological and orthographic cognitive processes. For example, attentional difficulties in children with ADHD could cause inefficiency with the trade-off between processing and storage functions of working memory during the learning to read (Daneman and Carpenter, 1980). If this were true, lower performance of children with ADHD in word recognition and spelling tasks might be attributed in part to the representations of distinct constructs, giving rise to differential functioning of the of the respective stimuli items. Therefore, it is essential to test measurement invariance to demonstrate that the observed differences in reading single words aloud and spelling under dictation are related to true mean differences as opposed to the instrument failing to capture equivalently these constructs between children with and without ADHD.

Two methods are frequently used for accessing measurement invariance, namely, Multigroup Confirmatory Factor Analysis (MGCFA) and Multiple Indicators Multiple Causes (MIMIC) modeling (Brown, 2015). In the first case, a theoretical model is compared with the observed structure of a certain measure in two or more samples and a series of constraints are made to the model. For example, if we assume that reading single words aloud presents a unidimensional factor structure, and we want to test if different groups of children (e.g., with and without ADHD) represent the construct of recognizing words at the same way, we can compare the theoretical model with the observed data for both groups, which is called configural invariance. If this condition is satisfied, we can test if the items of the word recognition task present similar factor loadings (“difficulty”) in both groups (i.e., metric invariance). Finally, we can test if children with ADHD who have the same level of reading aptitude as children without ADHD have the same probability to endorse items in a word recognition task (i.e., items present the same discrimination in both groups), which can be tested through scalar invariance.

The MIMIC modeling uses a unique matrix of data (e.g., word recognition and spelling scores) and characteristics of the subpopulations (e.g., diagnostic category) are regressed on the items that comprise the scale. If the subpopulation characteristic produces an effect on the individual items of the scale, it is an evidence of measurement non-invariance. Therefore, the items affected by the subpopulation characteristic are assumed to be affected by differential item functioning (DIF) and should be revised for diagnostic purposes because the items work differently in different subpopulations confounding the estimates generated by the scale. In other words, demonstrating DIF in a task implies that a given item could be easier (or more difficult) for a person based on his or her diagnostic category, regardless of his or her latent trait or actual ability.

Measurement invariance has been a topic of interest in clinical ADHD studies; however, most of those studies explored the underlying construct of ADHD across various subgroups defined by demographics, for example, based on gender, age, or different raters (Gomez, 2013, 2016; Makransky and Bilenberg, 2014; Narad et al., 2015; Zeeuw et al., 2015; Caci et al., 2016; Morin et al., 2016). In general, these studies demonstrated that ADHD is largely measurement invariant across gender, age, and raters. Few studies have investigated measurement invariance for reading measures (Furnes and Samuelsson, 2011; Pae et al., 2012; Cirino et al., 2013; Oliden and Lizaso, 2013; Farrington and Lonigan, 2015) and fewer still for spelling (Furnes and Samuelsson, 2011). Cirino et al. (2013) found out measurement invariance across groups of struggling and typical readers in a decoding task and partial measurement invariance for reading comprehension and fluency tasks. Farrington and Lonigan (2015) demonstrated DIF for few items of the Revised Get Ready to Read! Test, which is composed by phonological awareness and print knowledge tasks. All the effect sizes of items with DIF were small in magnitude and were related to age (older versus younger children) and race (African America versus White children). Oliden and Lizaso (2013) showed measurement invariance across five different languages (Catalan, Basque, Spanish, Galician, and Valencian) for the 2009 version of the reading comprehension test of the Program for International Student Assessment (PISA). Pae et al. (2012) compared struggling and typical adult readers in the Peabody Picture Vocabulary Test (3rd Edn) and did not found evidences for DIF across ability, gender, and age for the items of the receptive vocabulary knowledge task. Finally, Furnes and Samuelsson (2011) demonstrated that reading (measured by sight word reading and the phonological decoding) and spelling (single words and pseudowords) tasks were measurement invariant across orthographies (Norwegian/Swedish vs. English).

To our knowledge, no study has examined the possibility of measurement invariance for measures of word recognition and spelling between the subpopulations of children with and without ADHD. This question is essential to establish direct comparability of those subpopulations, and it is the main objective of the present study. The comparability of groups in a certain psychological trait should not be assumed; instead, it must be empirically demonstrated. The question addressed in the present study is relevant regarding the validity of word recognition and spelling measures and the use of these measures to compare populations of children with and without ADHD. This study will determine if the items composing word recognition and spelling single words tests measure the same underlying constructs (i.e., a unidimensional factor structures for each ability), and if these constructs exhibit similar relationships (i.e., similar factor loadings of the items on the latent variables) in the groups of children with and without a diagnosis of ADHD. Furthermore, we investigate if there is a significant direct effect of an ADHD diagnosis on the items that compose the reading and spelling tests, to determine if the items function differently between subgroups. We hypothesize that ADHD will not interfere with the constructs of word recognition and spelling and that the items will function comparably between the tasks, because the constructs underlying these abilities are tied to phonological representations of the words, rather than to domains of function (e.g., prefrontal functions such as attention and impulse control) affected in ADHD.

## Materials and Methods

### Participants

This study makes use of data from the baseline wave of a large longitudinal community school-based study from Brazil from which detailed methodological description is available (Salum et al., 2015). The sample came originally from 64 schools in grades 2–9 in the cities of São Paulo and Porto Alegre, Brazil. From a population of 12,500 families that were potential cases in the registry day at schools, 8,012 produced valid screening interviews. The 4488 families were excluded due refuse of participation, school registry not performed by a biological parent, incomplete screening interview, and other reasons such as changing school at the time of the evaluation or giving an invalid phone contact. Therefore, 8,012 families were contacted by phone or face-to-face to answer the Family History Survey (FHS; Weissman et al., 2000) to provide information about their 9,937 children. From this sample, we recruited two subgroups of participants: high-risk (*n* = 2,371) and random (*n* = 1,500) sample groups. The selection for the high-risk group was based on a risk prioritization procedure to identify individuals with current symptoms and/or a family history in five target disorders [ADHD, anxiety, obsessive-compulsive disorder (OCD), psychosis, and learning disorders]. Therefore, this procedure do not produce a diagnosis for these disorders, but create an index for screening possible risk for such target disorders. For example, for learning disorders screening, there were only two questions: “in childhood, somebody in your family presented difficulties with reading, writing, or math?” and “in the childhood, somebody in your family presented difficult for speaking or understanding what is said?” Based on this screening procedure, we lost 817 participants of the high-risk sample and 542 children from the random sample due withdrawal of participation, changing address, lost contact, and other reasons. Therefore, 1,554 children with high-risk for mental disorders and 958 children from the random sample were selected for further individual evaluation (*n* = 2,512). From this sample, we excluded those who did not complete all tasks, resting 2,401 children. For the purpose of the present study, we excluded the children with low intelligence quotient (IQ < 70), and those not yet instructed in reading and spelling (i.e., those in first grade as per the curriculum in Brazil). The final sample was therefore composed of 1,935 participants from 58 schools (77.03% of the 2,512 children; 61.34% from the high-risk group).

### Measures

#### Estimated IQ

IQ was estimated from scores on Vocabulary and Block Design subtests of the Wechsler Intelligence Scale for Children (WISC-III), using Tellegen and Briggs (1967) method. Residual associations with age were regressed out using Studentized residuals.

#### Psychiatric Diagnoses

Psychiatric diagnoses were derived from the Development and Well-Being Assessment (DAWBA; Goodman et al., 2000). DAWBA is a DSM and ICD based structured interview composed of verbatim and structured questions about the common emotional, behavioral, and hyperactivity disorders. For the present study, we evaluated the answers to the ADHD section of the instrument to assess inattention and hyperactivity/impulsivity in the whole sample (*n* = 1,935). DAWBA presents good evidence of validity and reliability (ω > 0.70 and EVC > 0.77 for the general factor; Wagner et al., 2016) and fair agreement with Child and Adolescent Psychiatric Assessment (CAPA) and the Diagnostic Interview Schedule for Children (DISC) (respectively, 0.49 and 0.57, according to Angold et al., 2012), making it suitable for epidemiological studies. The scale is composed of 18 items that evaluate ADHD symptoms using a Likert scale of three points (0–2) representing, respectively, the strength of the symptomatology, i.e., ‘No more than others,’ ‘A little more than others,’ and ‘A lot more than others,’ respectively. Based on the results, 212 children met full ADHD DSM-IV diagnose (77 predominantly inattentive; 28 predominantly hyperactive/impulsive; 79 combined type; and 28 other type). Therefore, 1,723 children did not present diagnostic criteria for ADHD, from which 958 were from the randomly selected sample and 765 were from high-risk sample.

#### Word Recognition and Spelling Assessment

To evaluate word recognition and spelling ability, we used the reading and spelling subtests of the School Performance Test (Stein, 1994). The School Performance Test is a basic academic test for children and adolescents with evidences of validity and reliability, with Cronbach’s alpha varying from 0.75 to 0.85 for each scale (e.g., Athayde et al., 2014; Lúcio and Pinheiro, 2014; Giacomoni et al., 2015). In the reading subtest, the children read aloud 70 isolated words presented on cards. In the spelling subtest, children write under dictation 34 isolated words selected from sentences. In both subtests, correct responses receive a score of 1.0 and incorrect responses receive a score of 0.0.

### Procedures

Each child was individually tested in a quiet room at their school or at their homes. Parents (87.7% mothers) responded to the DAWBA for psychiatric diagnosis of the children. All procedures followed standard instructions in the manuals (see section “Measures”).

### Ethics Statement

The research received approval from the Ethical Committee of the Federal University of São Paulo (protocol no. 1.327.777/15). The parents or legal guardians of all non-adult participants provided written informed consent prior to participation of their children, as well as written informed consent for their own participation. The children, in turn, gave their verbal assent to the evaluators.

### Statistical Analysis

Measurement invariance was tested using two procedures: MGCFA and MIMIC models. In both cases, the models were estimated with Mplus 7.0 (Muthén and Muthén, 2012) using a weighted least squares estimator (WLSMV).

For the CFA models, grouping was based on ADHD diagnostic status (ADHD vs. controls). To avoid bias due to sample selection (which is not randomly assigned, but based on high-risk), weights were created to counterbalance bias selection as described in detail elsewhere (Martel et al., 2016). Additionally, two multilevel features were added in the model to account for bias arising (a) children nested in schools (this is solved by the Mplus’ CLUSTER in VARIABLE command) and (b) an unequal probability of children selection via FHS’s variables enriching the frequency of both child and family psychopathology; more details can be found at Salum et al. (2015). To adjust for the latter (solved by WEIGHT in VARIABLE command), we used sample weights constructed using propensity score matching. For details, see Martel et al. (2016). Cohen’s *d* was used to estimate the effect size in the difference between reading and spelling abilities between children with and without ADHD.

#### Multigroup Confirmatory Factor Analysis

After proving evidence regarding the unidimensional fit of the word recognition and spelling tasks (initial model), a sequential strategy for testing measurement invariance was performed, following Meredith’s (1993) recommendations. This procedure determines if the meaning of the construct (i.e., discrimination of the items), and the difficulty of each individual item was equivalent across groups of children with and without ADHD. These criteria for configural and scalar invariance must be met in order to compare the groups on the latent variable. Because MGCFA involves two separate input matrices, constraining, in item response theory terms, the discrimination (called as *a*) and difficulty (called as *b*) parameters in both groups, it is likely to obtain bivariate empty cells.

Empty cells generate statistically perfect correlations between two items, meaning that they are not statistically distinguishable and, for purpose of the analysis, one or both should be removed. This problem is most common when variables have extreme cuts like (95% of children had correct answer for a given item against 5% who did not). Hence, where empty cells appeared, we excluded one of the items to maintain the maximum as possible number of original items for both reading and spelling tasks.

First, it was tested if the factor structure was the same between groups (i.e., configural invariance). For model fit and adjustment index, we report the chi-squared statistic (χ^2^), the 90% CI of the root mean square error of approximation (RMSEA), the comparative fit index (CFI), and the Tucker-Lewis Index (TLI). To interpret these indices, we follow the recommendations of Hu and Bentler (1999), and of Yu (2002): an adequate model fit is indicated by *p* > 0.05 for the χ^2^, RMSEA ≤ 0.06, CFI ≥ 0.95, and TLI ≥ 0.95. It was then tested if the item thresholds and factor loadings were equivalent between groups (i.e., scalar invariance).

To provide evidence of scalar against configural invariance, we systematically tested if imposing restrictions (i.e., if the discrimination and difficulty parameters are equal across children with and without ADHD) did not worsen the model as compared to the least constrained model wherein the parameter was freely estimated (Chen, 2007). Because the χ^2^ statistic is highly sensitive to sample size, we consider that the added restrictions do not worsen the model if ΔCFIs of the free and constrained models differ by less than 0.01 (Cheung and Rensvold, 2002), and if the change in RMSEA between models is less than 0.015.

#### Multiple Indicators Multiple Causes (MIMIC) Models

**FIGURE 1:**
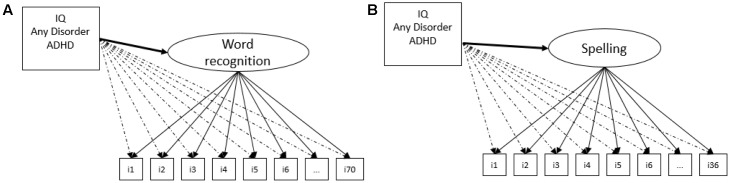
Multiple Causes (MIMIC) model for the word recognition **(A)** and spelling tasks **(B)**, illustrating the effects of the covariates on the general factor underlying the word recognition and spelling tasks (population heterogeneity) and the DIF test for the items (dashed lines from the covariates to the items).

Two different MIMIC models were conducted for word recognition and spelling measurement models: one using ADHD diagnostic (dichotomous variable status) and another incorporating ADHD symptoms as a continuous variable. Because MIMIC models do not split the sample (based on group comparisons), using a single covariance matrix, it does not require a large sample size compared to multiple-groups CFA (Brown, 2015). MIMIC is also called a CFA with covariates and it accommodates continuous covariates, whereas MCCFA only deals with categorical measures. As opposed to MGCFA, MIMIC only evaluates item thresholds and factor means as potential sources of invariance. A significant direct effect of the covariate (i.e., ADHD status or ADHD symptoms) on a reading or spelling item was taken as evidence of measurement non-invariance (an index of DIF) whereas a direct effect of the covariate on the latent variable is interpretable as evidence of population heterogeneity (i.e., group differences on factor means). In the MIMIC model, we added other two covariates concomitantly with ADHD as showing in the **Figure 1**: the IQ and a single dichotomous indicator of general psychopathology, which, via DAWBA, assess if the children endorse at least one positive item for the five domains of evaluated psychopathology (see section “Participants”), called here “any symptom of mental disorder.” As described in Brown (2015, p. 282), and here tested, “… [MIMIC] is frequently evaluated in an exploratory fashion.” We fixed all direct effects between ADHD, IQ, and the presence of any symptom of mental disorder to the 70 word recognition items and the 36 spelling items at zero. Then, it was inspected modification indices to determine if relevant direct effects would be presented. A modification indices > 4 of the covariate on an item presents DIF.

## Results

### Multigroup Confirmatory Factor Analysis (MGCFA)

**Table 1** presents the descriptive statistics for the study participants (means and standard deviations for age and IQ variables and proportion of female for the groups and for the total sample). The ADHD and control groups did not differ by age [*t*(1933) = 1.385, *p* = 0.166]. Nevertheless, the ADHD group presented significantly lower estimated IQ [*t*(1933) = 3.061, *p* = 0.002] and more males [χ^2^(1) = 5.461, *p* = 0.019], what agrees with previous results from the literature (e.g., Gershon and Gershon, 2002; Jepsen et al., 2009). **Table 2** presents the descriptive statistics of the raw scores for the word recognition and the spelling tasks, which were used for building the initial (baseline) model. Two items were excluded when testing the reading unidimensional model due to bivariate empty cells. The spelling model was admissible with all items included.

**Table 1:** Means and standard deviations (in brackets) for the variables age and IQ and proportion of females for the groups and the total sample.

Group	Age	IQ	Female
Control	9.98 (1.89)	101.25 (15.34)	48%
ADHD	9.64 (1.74)	97.84 (14.92)	40%
Total	9.80 (1.87)	100.87 (15.33)	47%

*IQ, estimated IQ; ADHD, attention deficit/hyperactivity disorder group.*

**Table 2:** Values of minimum, maximum, mean, and standard deviation for raw scores in the word recognition and spelling tests.

Group	*N*	Minimum	Maximum	Mean	*SD*
**Reading (raw score; 68 items)**					
Control	1719	0.00	68.00	53.66	19.16
ADHD	212	0.00	68.00	47.26	22.41
Total	1931	0.00	68.00	52.96	19.64
**Spelling (raw score; 34 items)**					
Control	1723	0.00	34.00	19.12	9.81
ADHD	212	0.00	33.00	15.79	9.83
Total	1935	0.00	34.00	18.76	9.87

*SD, standard deviation; N, sample size; ADHD, attention deficit/hyperactivity disorder group.*

For the initial measurement invariance models, both the reading and spelling models showed good fit indices for a unidimensional solution. For the initial reading model, mean item discrimination was 2.76 (*SD* = 1.40; minimum = 1.25; maximum = 7.38) and mean item difficulty was -0.87 (*SD* = 0.35; minimum = -1.52; maximum = -0.02). For the spelling initial model, mean item discrimination was 1.47 (*SD* = 0.64; minimum = 0.54; maximum = 4.35) and mean item difficult was -0.15 (*SD* = 0.67; minimum = -1.46; maximum = 1.34).

As a first step to investigate measurement invariance of the tasks, we tested the configural invariance, i.e., if the basic model structure is invariant across the groups (ADHD vs. controls). **Table 3** presents the tests of measurement invariance for the reading and spelling tasks. For the reading task, 30 out of the 68 original items were excluded due to bivariate empty cells. This model contained, therefore, 38 items in the word recognition task. For the spelling task, five items were excluded and the final model contained 29 items. For both tasks, configural invariance was achieved, meaning that the constructs measured by the tasks are unidimensionally represented in both groups.

**Table 3:** Model fit information for the reading and spelling tasks.

	*χ*^2^(*df*)	CFI	TLI	RMSEA	90% C.I.
**Model fit for the reading task (38 items)**					
Configural	1398.87 (1330)^a^	0.999	0.999	0.007	[0.000,0.011]
Scalar	1435.40 (1366)^a^	0.999	0.999	0.007	[0.000,0.011]
**Model fit for the spelling task (29 items)**					
Configural	900.73 (754)^a^	0.993	0.993	0.014	[0.010,0.018]
Scalar	928.16 (781)^a^	0.993	0.993	0.014	[0.010,0.014]

*^a^p < 0.05; χ^2^ = chi-square. CFI, comparative fit index; TLI, Tucker-Lewis Index; RMSEA, root mean square error of approximation; CI, confidence interval.*

As the configural model produced good fit index for both tasks, we investigated scalar invariance by holding the items’ factor loadings and thresholds (under item response theory called discrimination and difficulty, respectively) equal between the groups. For the word recognition and the spelling tasks, all items proved to be invariant (**Table 3**). Scalar against configural invariance was achieved for both reading and spelling tasks [reading: χ(36)^2^ = 43.489, *p* = 0.1827; spelling: χ(27)^2^ = 37.795, *p* = 0.0812]. For both models, ΔCFI was 0.000. Since scalar invariance was achieved, the mean in the latent traits can be compared in both groups. In the MGCFA models, word recognition and spelling abilities were poorer in children with ADHD as compared to children without, with a moderate effect size in the word recognition task (0.511, *p* < 0.0001) and small effect size in the spelling task (0.326, *p* = 0.004).

### MIMIC Modeling

**Figure 1** depicts the theoretical model for the MIMIC modeling for the word recognition (a) and the spelling tasks (b). **Table 4** presents model fit index for the MIMIC analysis. Regardless if ADHD was measured as continuous or dichotomous covariate, the four MIMIC models showed excellent fit index. The MIMIC analysis confirmed the absence of DIF for all items in both tasks regardless of the ADHD diagnostic status or inattention and hyperactivity impulsivity scores, IQ, and the presence of any symptom of mental disorder. Regarding population heterogeneity, ADHD as continuous variable predicted poorer word recognition (β = -0.195, *p* < 0.001) and spelling (β = -0.15, *p* < 0.001) latent traits. IQ predicted positively word recognition and spelling (β = 0.249, *p* < 0.001 and 0.252, *p* < 0.001, respectively). In the MIMIC models, an ADHD diagnosis was associated with poorer word recognition (Cohen’s *d* = 0.371, *p* = 0.001) and spelling (Cohen’s *d* = 0.349, *p* = 0.003) abilities. Lack of evidences were found for population heterogeneity in relation to the presence of any symptom of mental disorder for both word recognition (Cohen’s *d* = -0.051, *p* = 0.512) and spelling (Cohen’s *d* = -0.055, *p* = 0.367) tests.

**Table 4:** Model fit information for the MIMIC models for reading and spelling latent traits with ADHD symptoms (raw scores on DAWBA) and ADHD diagnostic status as covariates.

	*χ*^2^(*df*)	CFI	TLI	RMSEA	90% C.I.
**Models fit for the reading task (70 items)**					
ADHD symptoms	2653.830 (2411)^a^	0.998	0.998	0.007	[0.005,0.009]
ADHD diagnostic status	2661.894 (2411)^a^	0.998	0.998	0.007	[0.005,0.009]
**Models fit for the spelling task (34 items)**					
ADHD symptoms	771.762 (626)^a^	0.992	0.992	0.011	[0.008,0.013]
ADHD diagnostic status	772.044 (626)^a^	0.993	0.992	0.011	[0.008,0.013]

*^a^p < 0.05; χ^2^ = chi-square. CFI, comparative fit index; TLI, Tucker-Lewis Index; RMSEA, root mean square error of approximation; CI, confidence interval; ADHD, attention deficit/hyperactivity disorder; DAWBA, Development and Well-Being Assessment; MIMIC, Multiple Indicators Multiple Causes.*

## Discussion

The present study investigated measurement invariance in word recognition and spelling measures (reading aloud and spelling isolated words) for groups of children with and without ADHD in a sample of school-aged children. To our knowledge, this is the first study to evaluate measurement invariance for word recognition and spelling latent traits considering the ADHD diagnostic status as subpopulations in a large community based sample. Two structural equation modeling techniques were used to investigate and confirm the results. In multigroup CFA, properties of configural, scalar, and scalar against structural invariance were demonstrated for both word recognition and spelling tests. In MIMIC models (**Figure 1**), no evidence of DIF was found based on ADHD diagnostic status or ADHD symptoms (as a continuous variable representing). This last result was obtained even after controlling for IQ and the presence of any symptom of mental disorder. The results indicate that word recognition and spelling scores can be compared between children with and without ADHD, regardless of the severity of inattention and hyperactivity-impulsivity domain symptoms. In addition, it provides support that lower performance in word recognition and spelling in children with ADHD, when compared to children without ADHD, are not due to measurement problems.

In the present study, under MGCFA, ADHD children were 0.51 SD below children without ADHD in the word recognition latent trait and 0.33 SD lower in the spelling latent trait. Based on the invariance measurement results, it is safe to conclude that these differences are true differences between the groups, and not merely artifacts of the task performing differently between groups. Therefore, the results of this study endorse and confirm previous results indicating lower scores obtained by children with ADHD in reading and spelling tasks in relation to unaffected children (e.g., Willcutt et al., 2005; Greven et al., 2012; Johnels et al., 2014; Re et al., 2014; Pham, 2016; Miranda et al., 2017).

Our DIF analysis in MIMIC models was also used to confirm the previously obtained results with MGCFA, determining the extent to which item properties were influenced by characteristics of the children. No evidence of DIF was found for the items of the word recognition and the spelling tasks, as the results did not change considering both diagnostic status (i.e., children with vs. without ADHD) and dimensional inattention and hyperactivity-impulsivity scores. These results confirm that children with different levels of ADHD symptoms or those reaching a threshold to be diagnosed with ADHD vs. those without a diagnosis, have equal probabilities to endorse correctly the items in the word recognition and spelling tasks.

Demonstrating measurement invariance between groups in a measure is important to avoid bias that could invalidate comparisons between these groups. When equivalence is not attested, subjects with the same level of competence (ability or latent trait) can attain different scores in the measure, leading to erroneous conclusions about means differences. As states Chen (2008), “meaningful comparisons of statistic, such as means and regressions coefficients, can only be made if the measures are comparable across different groups” (p. 1005). Therefore, when measurement invariance is not achieved, two outcomes are probable: the group differences discovered in the study could be measurement artifacts; or true mean differences could be hidden by these very same artifacts. Widaman and Reise (1997) recommend a conservative approach to avoid problems due measurement non-invariance in the data, i.e., measurement invariance should be tested as a first step in research that uses group comparisons. It would avoid that our clinical interpretations about groups are made upon measures that “compare chopsticks with forks” (Chen, 2008). When measurement invariance is not achieved, the researcher may test different approaches, such as eliminate non-invariant items or using a partial measurement invariance model (Chen, 2008); in our study, some items in MGCFA were excluded; however, such exclusion was not due to invariance. Bivariate empty cells were among some items that appeared as consequence of MGCFA procedure *per se* where the sample is split and the correlation matrix, per group, is estimated. The other adopted measurement invariance technique, MIMIC, allowed us to verify issues related to DIF, and because MIMIC is not so restrictive (e.g., sequence of constrained parameters across the groups) regarding the process involving to invariance testing, we observed that in terms of difficulty, none of the items showed DIF.

The results for both CFA and MIMIC models confirmed measurement invariance and, therefore, the direct comparability of the groups in such tasks. Nevertheless, some limitations of the study should be acknowledged. First, diagnostic status for ADHD was assessed based only in a structured interview (i.e., DAWBA) administered to biological parents, as opposed to psychiatric assessments of the children directly or by including evaluations by teachers. Second, although weights were used to minimize selection bias in the high-risk study, the data from this community sample may not generalize to predominantly clinical populations. Third, despite we demonstrated measurement invariance above and beyond the influence of IQ and the presence of any symptoms of mental disorders, we still have to demonstrate that the presence of learning disorders will not affect the results. Finally, the extent that our results are limited to word recognition and spelling single words, or are applicable to other related abilities, as reading comprehension and expressive writing (which are also commonly to be lower in children with ADHD), remains to be demonstrated (e.g., Re et al., 2007; Martinussen and Mackenzie, 2015).

## Conclusion

The results of this study suggest that the domains of function affected in ADHD do not alter the constructs of word recognition and spelling abilities, which are tied to the processing of phonological and orthographic representations. ADHD symptoms do not change the probability to correctly endorse items in the used tests and children with ADHD do not consider the word recognition and spelling items more difficult than children without ADHD given a comparable amount of latent word recognition and spelling ability. On the other hand, children with ADHD have lower mean performance, demonstrated by the contrast on both trait levels. Therefore, ADHD symptoms (or the domains affected in the disorder, such as attention) may lead to poorer word recognition and spelling achievement, but this is not due to changes in how the construct is being measured. For clinical practice, it means that specific norms for scoring word recognition and spelling tasks are not recommended for children with ADHD because they are directly comparable to children without ADHD. Implications for education of children with ADHD may indicate that teaching practices should involve more the management of the ADHD symptoms (e.g., inattention for executing the word recognition/spelling tasks) than specificities in the teaching procedures itself (i.e., differences in teaching methodologies for this group of children). Given that the direct effect of ADHD on reading/spelling skills is not biased due to DIF, although these traits are highly correlated, it is suggested that identifying the neuropsychological deficits common to children with reading/spelling difficulties and ADHD might clarify the nature of high co-prevalence of these disorders (e.g., Willcutt et al., 2010; Lúcio et al., 2017).

## Author Contributions

The research is part of the doctoral dissertation^∗^ of PL, who conceived the research question, analyzed and interpreted the data, and drafted and reviewed the manuscript considering coauthors directions. HC-M was the advisor and contributed with the analysis and interpretation of the data, and with theoretical and methodological insights. GS, JM, RB, and LR conceived, planned, and carried out the “High risk cohort study,” from which this research is part. The authors contributed with insights in the manuscript’s writing on the topic of ADHD and its relation to Reading/spelling disability and critically revised de manuscript. WS helped with analysis and interpretation of the data and revised the manuscript in terms of content and grammar. PP and AG participated of data collection, training the psychiatrists and the lay-interviewers of the study. They critically revised the article and provided important insights to data interpretation. All the authors declare agreement with the submitted version of the manuscript and ensure that questions related to the accuracy or integrity of any part of the work are appropriately investigated and resolved. ^∗^The dissertation represents the only medium it has appeared in, is in line with the author’s university policy, and cannot be accessed online.

## Conflict of Interest Statement

RB has received research grants from AstraZeneca, Janssen Cilag, Novartis, Roche and the governmental funding agencies: CAPES, CNPq, and FAPESP; has been a forum consultant for Eli Lilly, Janssen, Novartis, and Roche; and has participated in speaker bureaus for Ache, Janssen, Lundbeck and Novartis, in the last 5 years. Dr. Nuechterlein is a non-compensated officer of MATRICS Assessment, Inc., he has received unrelated research support from Janssen Scientific Affairs, Genentech, and Posit Science, Inc., and he has consulted to Genentech, Otsuka, Janssen, and Takeda. LR was in the speakers’ bureau, and acted as a consultant for Eli-Lilly, Janssen-Cilag, Novartis, and Shire in the last 3 years. He receives authorship royalties from Oxford Press and ArtMed. He also received travel award (air tickets + hotel) for taking part in two child psychiatric meetings from Novartis and Janssen-Cilag in 2010. The ADHD and Juvenile Bipolar Disorder Outpatient Programs chaired by him received unrestricted educational and research support from the following pharmaceutical companies in the last 3 years: Abbott, Eli-Lilly, Janssen-Cilag, Novartis, and Shire. The other authors report no conflicts of interest to disclose.
